# Does elevated atmospheric CO_2_affect soil carbon burial and soil weathering in a forest ecosystem?

**DOI:** 10.7717/peerj.5356

**Published:** 2018-07-27

**Authors:** Miquel A. Gonzalez-Meler, Armen Poghosyan, Yaniria Sanchez-de Leon, Eduardo Dias de Olivera, Richard J. Norby, Neil C. Sturchio

**Affiliations:** 1 Department of Biological Sciences and Department of Earth and Environmental Sciences, University of Illinois at Chicago, Chicago, IL, USA; 2 Space Center, Skolkovo Institute of Science and Technology, Moscow, Russia; 3 Department of Agro-environmental Sciences, Universidad de Puerto Rico at Mayaguez, Mayaguez, Puerto Rico; 4 Environmental Science Division and Climate Change Science Institute, Oak Ridge National Laboratory, Oak Ridge, TN, USA; 5 Department of Earth and Environmental Sciences, University of Delaware, Newark, DE, USA

**Keywords:** Soil C, Elevated CO_2_, Isotope, Temperate forest, Bioturbation, cesium-137, lead-210

## Abstract

Most experimental studies measuring the effects of climate change on terrestrial C cycling have focused on processes that occur at relatively short time scales (up to a few years). However, climate-soil C interactions are influenced over much longer time scales by bioturbation and soil weathering affecting soil fertility, ecosystem productivity, and C storage. Elevated CO_2_can increase belowground C inputs and stimulate soil biota, potentially affecting bioturbation, and can decrease soil pH which could accelerate soil weathering rates. To determine whether we could resolve any changes in bioturbation or C storage, we investigated soil profiles collected from ambient and elevated-CO_2_plots at the Free-Air Carbon-Dioxide Enrichment (FACE) forest site at Oak Ridge National Laboratory after 11 years of ^13^C-depleted CO_2_ release. Profiles of organic carbon concentration, *δ*^13^C values, and activities of ^137^Cs, ^210^Pb, and ^226^Ra were measured to ∼30 cm depth in replicated soil cores to evaluate the effects of elevated CO_2_ on these parameters. Bioturbation models based on fitting advection-diffusion equations to ^137^Cs and ^210^Pb profiles showed that ambient and elevated-CO_2_ plots had indistinguishable ranges of apparent biodiffusion constants, advection rates, and soil mixing times, although apparent biodiffusion constants and advection rates were larger for ^137^Cs than for ^210^Pb as is generally observed in soils. Temporal changes in profiles of *δ*^13^C values of soil organic carbon (SOC) suggest that addition of new SOC at depth was occurring at a faster rate than that implied by the net advection term of the bioturbation model. Ratios of (^210^Pb/^226^Ra) may indicate apparent soil mixing cells that are consistent with biological mechanisms, possibly earthworms and root proliferation, driving C addition and the mixing of soil between ∼4 cm and ∼18 cm depth. Burial of SOC by soil mixing processes could substantially increase the net long-term storage of soil C and should be incorporated in soil-atmosphere interaction models.

## Introduction

Soils contain most of the organic carbon in Earth’s “critical zone”, thus formation, transport and degradation of soil organic carbon (SOC) are key factors in the global carbon cycle (Hopkins et al., 2013). Fixation of atmospheric CO_2_ by plant photosynthesis and the consequent decomposition and release of this organic carbon as CO_2_ by soil biota are principal factors in the evolution of the SOC pool and the atmospheric concentration of CO_2_. Soil organic carbon decomposition depends on vegetation, microbial community, molecular composition of the organic matter, mineralogy, moisture, and temperature (Jastrow, 1996; Jastrow, Amonette & Bailey, 2007; O’Brien et al., 2010; Cheng et al., 2014). Climate change forcing factors can directly and indirectly affect soil C stocks, altering the resilience of vegetation and human society to climate change (Jastrow et al., 2005; Hungate & Hampton, 2012;  Gonzalez-Meler, Rucks & Aubanell, 2014;  Marshall & Gonzalez-Meler, 2016). However, the long-term fate of terrestrial soil C stocks under climate change scenarios may also be a function of soil C transport and burial processes (Chaopricha & Marin-Spiotta, 2014). Transport of SOC within the soil C matrix is difficult to measure but SOC burial has been recognized in playing a role in the responses of the soil C pool to climatic factors ( Lehmann & Kleber, 2015).

Mechanical mixing of soil by bioturbation (the mixing of soil particles by biological agents) can modulate the rate of SOC decomposition by vertical transport, potentially bringing SOC from the surface to depth, and vice-versa (Gabet, Reichman & Seabloom, 2003; Wilkinson, Richards & Humphreys, 2009). This process operates slowly and affects the SOC cycle on centurial time-scales, yet its effects must be taken into account when modeling carbon fluxes at regional or global scales (Koven et al., 2009; Drewniak & Gonzalez-Meler, 2017). Our need to understand the climate feedbacks caused by the alteration of the global carbon cycle is becoming more urgent because of the dramatic increase in atmospheric CO_2_ caused by anthropogenic activities. Long-term predictions of Earth system responses to global climate change or CO_2_ increase require a better understanding of soil C processes that operate at multi-decadal time scales (e.g., O’Brien et al., 2010; O’Brien et al., 2013; O’Brien et al., 2015) to model future biosphere feedbacks on atmospheric greenhouse gas composition. Specific mechanistic information on bioturbation in temperate forested ecosystems is limited (Fujiyoshi & Sawamura, 2004; Kaste, Heimsath & Bostick, 2007; Kaste et al., 2011), and available studies generally do not explicitly link long term soil C movement to climate change forcing factors.

Soil biota can alter soil chemical and physical properties in response to climate change and perhaps accelerate soil mixing and C burial rates (Wilkinson, Richards & Humphreys, 2009; Sánchez-de León et al., 2014; Chaopricha & Marin-Spiotta, 2014). Increased soil biological activity in ecosystems exposed to elevated CO_2_ often increases soil CO_2_ concentrations ( Gonzalez-Meler & Taneva, 2011), that may cause soil acidification and increased weathering rates (Andrews & Schlesinger, 2001;  Bernhardt et al., 2006). Lowering pH and increased plant nutrient uptake may result in loss of soil fertility, affecting the way plants further respond to elevated CO_2_. Evidence for net loss of metal and cations via leaching has been shown in some elevated CO_2_ studies (Cheng et al., 2010) but not in others (Oh et al., 2007; Kaste et al., 2011; Duval et al., 2013). The bulk of C and nutrients in the soil is associated with particles, yet it is not well understood how soil particle mixing would determine long-term C storage in a high-CO_2_ world.

The Free-Air Carbon Dioxide Enrichment (FACE) enrichment experiment at Oak Ridge National Laboratory (ORNL) in eastern Tennessee, USA, provided an opportunity to examine the effects of elevated atmospheric CO_2_ on SOC and bioturbation in a closed-canopy deciduous forest ecosystem (Norby et al., 1999; Norby et al., 2001). This site has been shown to accrue more soil C at the elevated CO_2_ conditions when compared to ambient conditions (Jastrow et al., 2005). In addition, elevated CO_2_ has enhanced root proliferation (Matamala et al., 2003; Iversen et al., 2011; Lynch et al., 2013) and earthworm activity (Sánchez-de León et al., 2014; Sánchez-de León et al., 2018), two major drivers of bioturbation in temperate forest soils (Wilkinson, Richards & Humphreys, 2009).

In conjunction with the soil C cycle and earthworm studies, we measured soil profiles of fallout ^137^Cs and ^210^Pb activities, along with those of ^40^K and ^226^Ra. Large pulses of ^137^Cs were introduced into the stratosphere during thermonuclear weapons tests of the 1950s and 1960s, with a well-defined maximum deposition at Earth’s surface occurring in 1963. This surface deposition of ^137^Cs and other weapons fallout radionuclides provides a globally distributed time horizon in soils and sediments, which has been used widely to determine sedimentation rates and sediment mixing by organisms in soils, lakes and oceans (Guinasso & Schink, 1975; Olsen et al., 1981; Robbins, 1986; Kaste, Heimsath & Bostick, 2007; Kaste et al., 2011). In contrast to the bomb-pulse input of ^137^Cs, ^210^Pb is continuously deposited from the atmosphere and is also produced by decay of ^226^Ra in soil via ^222^Rn. Because ^137^Cs and ^210^Pb are strongly adsorbed to soil particles and are not biologically transformed, they are especially useful as tracers of soil mixing and bioturbation at different shallow soil depths (Bruckmann & Wolters, 1994; Bunzl, 2002a; Bunzl, 2002b; Schuller et al., 2004; Kaste, Heimsath & Bostick, 2007; Kaste et al., 2011). In this study, we apply advection-diffusion models to estimate bioturbation rates from ^137^Cs and ^210^Pb profiles in soils of the ORNL FACE site, and use these results along with ^226^Ra and ^40^K profiles to compare bioturbation, redistribution of SOC, and potential weathering effects under ambient and elevated-CO_2_ conditions.

## Material and Methods

The CO_2_ treatment at the ORNL FACE experiment was initiated in 1998 and continued for 12 growing seasons through 2009. The site is contained in a 1.7-hectare sweetgum (*Liquidambar styraciflua* L.) plantation on the Oak Ridge National Environmental Research Park that was planted with 1-year-old trees in 1988 on an upland terrace of the Clinch River. The FACE experiment comprised five 25-m diameter plots (two elevated CO_2_ and three control plots), each plot representing a replicate. The CO_2_ concentration in the elevated CO_2_ plots was maintained about 150 ppm above ambient during the experiment, at first continuously until 2001, and then only during daylight hours through the end of the experiment in 2009. Soil at the ORNL FACE site is classified as an Aquic Hapludult (Ultisol) that developed from alluvium derived from dolomite, sandstone, and shale. It is a moderately well drained, slightly acidic, silty clay loam soil with high base saturation (Van Miegroet, Norby & Tschaplinski, 1994). Results of the ORNL FACE experiment have been highlighted in several articles (Matamala et al., 2003; Norby et al., 2010; Iversen et al., 2012).

### Sampling and sample preparation

Soil samples were collected from the ORNL FACE site ten years into the experiment in September 2008. We used a sharpened steel pipe (4.8 cm diameter) driven into the soil with a nylon-face mallet (Sánchez-de León et al., 2018) to obtain four cores from each of the ambient (control) plots and four from each of the elevated CO_2_ plots. Intact soil cores were stored frozen and sectioned with a thin ice-core saw (while frozen and the blades cleaned between cuts) as follows: the top 8 cm of each core was sectioned into 1-cm depth increments, and from 8 to ∼20 cm depth the core was sectioned into 2-cm depth increments. Additional soil core samples from 20 to 25 and 25 to 30 cm were collected adjacent to each sampling spot to help constrain the maximum depth of measurable ^137^Cs activity. No samples were collected below 30 cm depth for these experiments. We compared our samples with soils samples collected in 1997 (prior to the initiation of FACE experiment) and archived. Pre-treatment core samples were for depth ranges of 0–5, 0–15, 15–30, and 30–45 cm from both the ambient and elevated-CO_2_ plots.

After sectioning the soil cores, rocks and roots were manually removed from each section. Samples were dried at 80 °C, gently crushed and sieved to pass through a 2-mm sieve. Dry bulk densities were calculated from separate samples by comparing the 2 mm-sieved soil dry weight with the core section volume after correction for the occasional small rocks being removed.

### Soil organic carbon concentration and stable C isotope ratios

Soil samples were ground to a fine powder for analysis of organic C concentration and stable C isotope ratios. Carbonates were removed before analyses as explained elsewhere (O’Brien et al., 2015). Analyses were performed at the Ecology Stable Isotope Laboratory (UIC) using a Costech ECS 4010 elemental analyzer with a zero-blank autosampler interfaced with a ThermoFinnigan Delta-Plus XL isotope ratio mass spectrometer in continuous flow. Soil organic C concentrations are reported in %C (dry weight basis). The ^13^C/^12^C isotope ratios are reported in the conventional delta notation, in units of per mil relative to the standard reference material VPDB (Coplen, 1996), according to:

(1) \begin{eqnarray*}& & {\delta }^{13}\mathrm{C},\permil =[({R}_{\mathrm{ sample}}/{R}_{\mathrm{V PDB}})-1]\times 1,000\end{eqnarray*}

where *R* is the atom ratio ^13^C/^12^C. Reproducibility of *δ*^13^C values is better than ±0.1‰  when compared to international standards.

### Gamma spectrometry

Gamma spectrometry was performed at the Environmental Isotope Geochemistry Laboratory (UIC) by using a Canberra model GR3020 reverse-electrode intrinsic Ge detector system interfaced with a DSA-2000 digital spectrum analyzer. Dry homogenized sediment samples (5–10 g) were weighed into aluminum counting cans and these were sealed with Al foil. Gamma activities for ^40^K, ^137^Cs, ^210^Pb, and ^226^Ra were measured at 1,460.5, 661.6, 46.5, and 186.2 keV, respectively, with cans centered on top of the detector. Detector efficiency was calibrated versus sample weight in the same geometry using the certified standards CANMET DL-1a (U-Th ore diluted in quartz sand) and NIST SRM-4357 (Ocean Sediment). Relative uncertainties of measured activities were less than ±10% for activities >4 Bq kg^−1^, as calculated from counting statistics incorporating background subtraction and propagated errors. Activities were measured per sample dry mass and normalized to dry bulk density measurements for reporting in units of Bq cm^−3^ or Bq kg^−1^.

### Bioturbation model based on ^137^Cs profiles

Mathematical models combining advection and diffusion have been developed to explain the downward movement of ^137^Cs and the diffusion-like broadening of its profile in soils and sediments (Guinasso & Schink, 1975; Olsen et al., 1981; Robbins, 1986). The steady-state bioturbation model (Eq. (2) in Robbins, 1986) describes the total concentration [*C*(*x*, *t*)] of particle-bound radionuclides in the soil as a function of the vertical distance (*x*), and time (*t*). Biological agents and advection explain the downward transport of ^137^Cs (see Eq. (2)). The biodiffusion coefficient (*D*_*b*_) describes diffusive mixing of bulk soil by biological agents. Transport of ^137^Cs can also be caused by advective processes involving motion of particles and pore fluid (*ν*). The net loss or gain of the ^137^Cs within the soil profile is accounted for by the radioactive decay constant (*λ*) and the first-order feeding rate constant that describes net transport of ^137^Cs by moving organisms (*γ*):

(2) \begin{eqnarray*}& & \frac{\partial C}{\partial t} = \frac{\partial }{\partial x} \left( {D}_{b} \frac{\partial C}{\partial x} \right) - \frac{\partial }{\partial x} \left( \nu C \right) - \left( \lambda + \gamma \right) C.\end{eqnarray*}

Best-fits for the average ambient and elevated-CO_2_^137^Cs profiles used the following fixed values for the model (Eq. (2)): 0.75 Bq cm^−2^ for the initial activity of ^137^Cs (C_0_) (Hardy et al., 1968; CDC-NCI, 2005); 45 years for time elapsed (*t*) between ^137^Cs tracer deposition in 1963 and sample collection in 2008 (or 34 years for 1997); and, 0.023 yr^−1^ for the ^137^Cs decay constant (*λ*). For a pulse-like input of the tracer, the model represented in Eq. (2) has a well-known solution, which has been widely used to describe ^137^Cs profiles in soils (Van Genuchten & Cleary, 1979; Ivanov et al., 1997; Bossew & Kirchner, 2004; Schuller et al., 2004).

(3) \begin{eqnarray*}& & C \left( x,t \right) ={C}_{0}{e}^{-(\lambda + \gamma )t} \left\{ \frac{1}{\sqrt{\pi {D}_{b}t}} {e}^{-{ \left( x- \nu t \right) }^{2}/(4{D}_{b}t)}- \frac{\nu }{2{D}_{b}} {e}^{\nu x/{D}_{b}} \text{erfc} \left( \frac{x+\nu t}{2\sqrt{{D}_{b}t}} \right) \right\} .\end{eqnarray*}

We calculated error-weighted least-squares best fits of Eq. (3) to the data for each of our measured ^137^Cs profiles by using MATLAB.

### Bioturbation model based on unsupported ^210^Pb profiles

Mathematical models combining advection and diffusion terms to account for downward transport and dispersion of unsupported ^210^Pb differ from those for ^137^Cs, because ^137^Cs is deposited in a pulse-like manner whereas ^210^Pb is deposited continuously as it is produced from decay of atmospheric ^222^Rn (Robbins, 1978). We used the following steady-state equation to describe advective-diffusive transport of ^210^Pb (Kaste et al., 2011), where *A*_*Z*_ is the initial activity of unsupported ^210^Pb at the surface (Bq/kg); *A*_*Z*_ is the activity of unsupported ^210^Pb at depth *z* (cm); *v* is the advection rate

(4) \begin{eqnarray*}& & A(z)={A}_{0}~\mathrm{exp} \left[ \frac{\nu -\sqrt{{\nu }^{2}+4\lambda D}}{2D} (z) \right] .\end{eqnarray*}

(cm yr^−1^); *D* is the diffusion constant (cm yr^−1^); and *λ* is the decay constant of ^210^Pb (0.031 yr^−1^).

## Results

### Soil organic carbon and *δ*^13^C profiles

**Figure 1:**
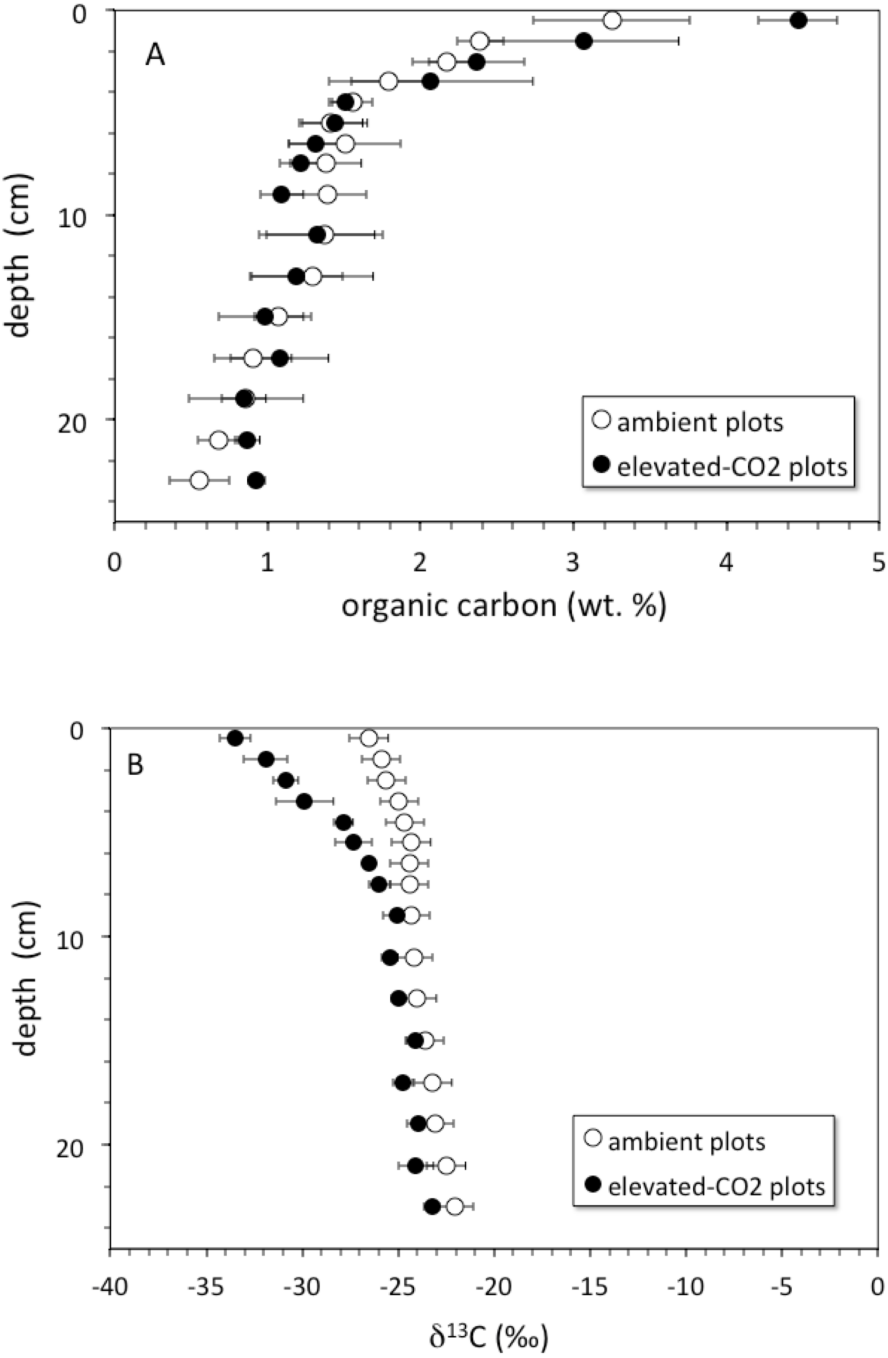
Soil organic carbon vs depth. (A) depth (cm) vs. soil organic carbon (wt. %) for average core samples from ambient (open circles) and elevated-CO_2_ plots (filled circles); (B) depth (cm) vs. *δ*^13^C (‰) for average core samples from ambient (open circles) and elevated-CO_2_ plots (filled circles).

**Figure 2:**
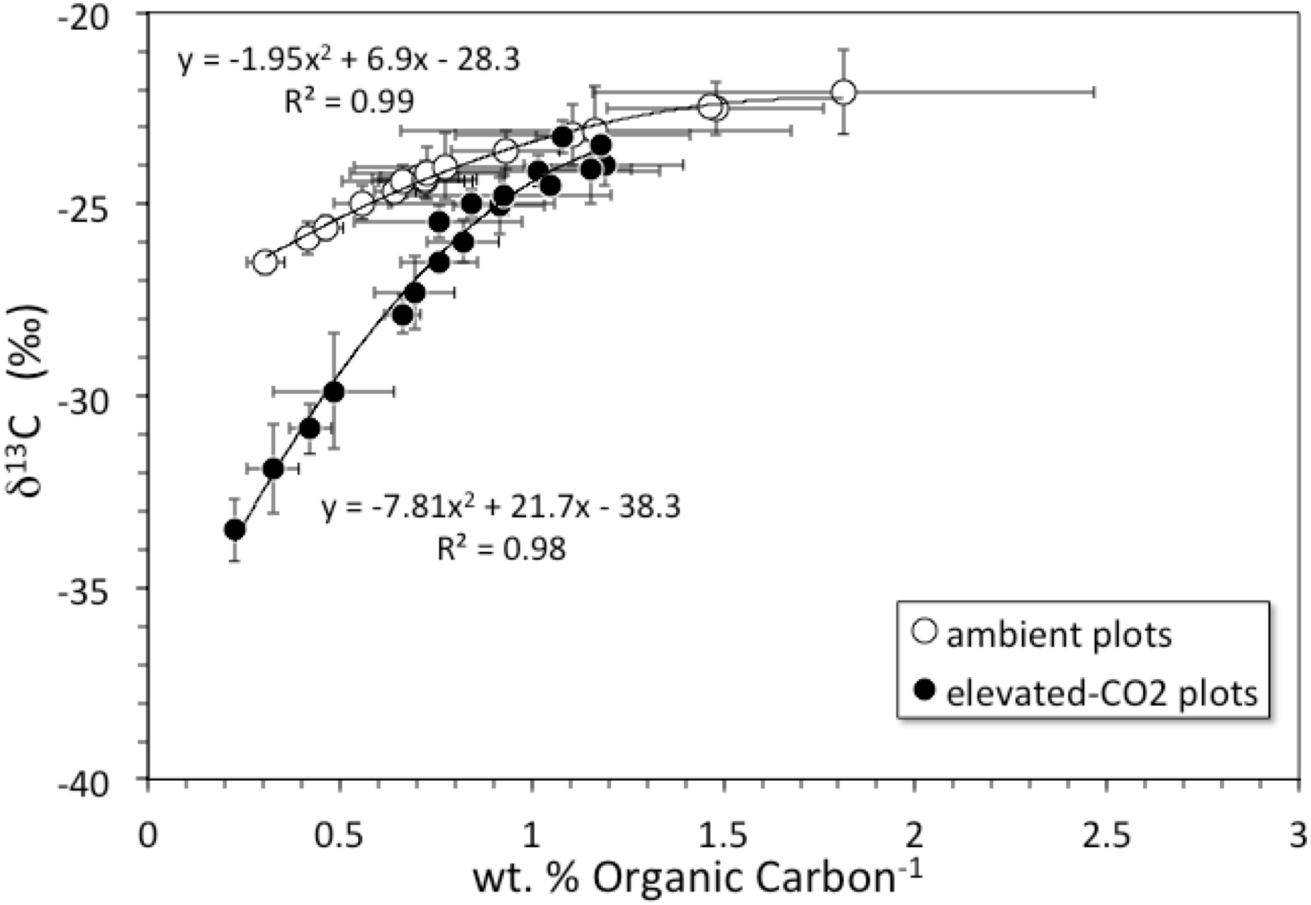
*δ*^13^C vs. inverse concentration of organic carbon. Diagram showing *δ*^13^C (‰) vs. inverse concentration of organic carbon (1/wt. %) for averages of core profiles from the ambient CO_2_ plot (open circles) and the elevated-CO_2_ plot (filled circles). Black lines are 2nd-order polynomial best fits. Shift of the elevated-CO_2_ profile toward the *Y*-axis indicates enrichment in organic carbon relative to the ambient CO_2_ profile. *Y*-intercepts represent contrasting *δ*^13^C values of organic carbon being added to the surface under ambient and elevated-CO_2_ conditions.

Soil organic carbon content was highest at the surface and decreased with depth (Fig. 1A). The top 6 cm of the elevated-CO_2_ profiles, on average, have *δ*^13^C values significantly lower than in the ambient profiles (Fig. 1B), as indication of SOC inputs since the initiation of the experiment in 1998. As a result of new inputs, the top 2 cm of the elevated-CO_2_ profiles, on average, have significantly higher SOC content than in the ambient profiles (*p* < 0.05), but below 2–4 cm depth the average profiles are not significantly different (Fig. 1A). When the *δ*^13^C values are compared with the inverse SOC content, it is apparent that the average elevated-CO_2_ profile is depleted in ^13^C. The SOC being deposited at the soil surface has a *δ*^13^C value of about −38‰  in the elevated-CO_2_ plot compared with −28‰  in the ambient plot (Fig. 2).

Soil bulk density was lower at the top 5 cm of the soil profile than at the rest of the soil depths (Table 1). Bulk density increased from values of about 0.5 g cm^−3^ at shallow depths to values greater than 1 g cm^−3^ at 5–6 cm depth and deeper. This may reflect degradation and mineralization of the litter layer which occurs during the first decades following deposition and produces denser residual material (Kaste et al., 2011).

**Table 1:** Soil bulk density (kg cm^−3^) across the soil profile for sections of soil cores collected at ambient and Elevated CO_2_ plots at the ORNL FACE experiment in 2008. Values are averages of three and two replicates for ambient and elevated plots, respectively, with standard errors.

**Soil depth (cm)**	**Ambient CO_2_**	**Elevated CO_2_**
0–1	0.58 ± 0.07	0.46 ± 0.07
1–2	0.83 ± 0.11	0.85 ± 0.01
2–3	0.97 ± 0.21	1.08 ± 0.31
3–4	0.96 ± 0.32	1.23 ± 0.25
4–5	1.16 ± 0.09	1.02 ± 0.01
5–6	1.27 ± 0.06	1.43 ± 0.13
6–7	1.43 ± 0.20	1.00 ± 0.19
7–8	1.26 ± 0.11	1.26 ± 0.22
8–10	1.16 ± 0.09	1.43 ± 0.16
10–12	1.30 ± 0.07	1.11 ± 0.09
12–14	1.26 ± 0.03	1.12 ± 0.20
14–16	1.36 ± 0.05	1.23 ± 0.12
16–18	1.28 ± 0.16	1.17 ± 0.04
18–20	1.28 ± 0.06	1.18 ± 0.12
20–25	1.46 ± 0.02	1.36 ± 0.08
25–30	1.16 ± 0.01	1.68 ± 0.05

### ^137^Cs profiles

**Figure 3:**
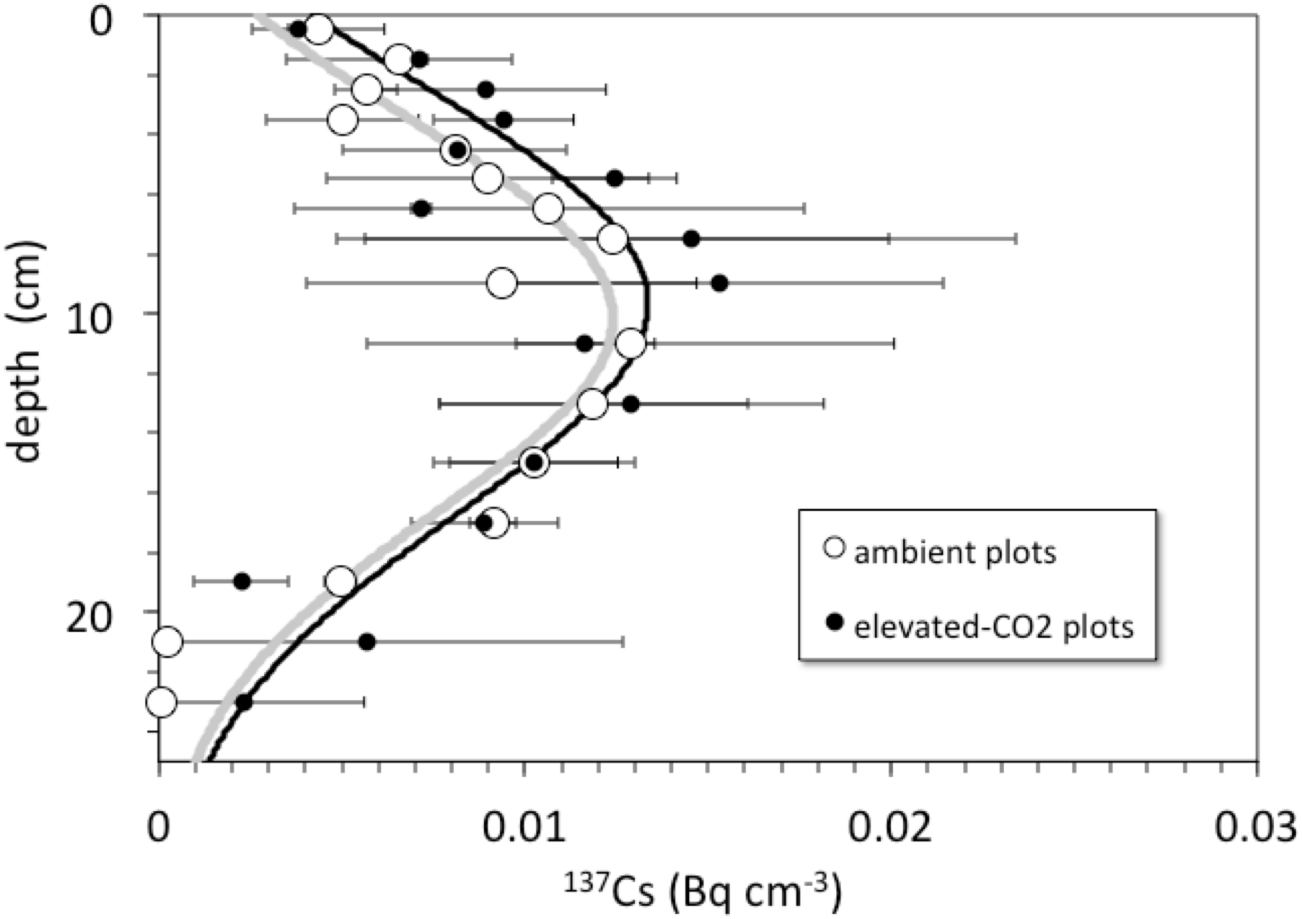
Depth vs. ^137^Cs activity. Depth (cm) vs. average ^137^Cs activity (Bq cm^−3^) in cores collected from the ambient (open circles) and elevated-CO_2_ (filled circles) plots at the Oak Ridge FACE site. Solid lines (gray, ambient; *R*^2^ = 0.84; black, elevated-CO_2_; *R*^2^ = 0.75) are best-fit advection-diffusion model profiles based on Eq. (2).

Detectable ^137^Cs was measured from the surface to a depth of at least 20–30 cm in all soil profiles (Fig. 3). Total ^137^Cs inventories of the soil profiles are less than or equal to that expected if the assumed initial activity of ^137^Cs (0.75 Bq cm^−3^) remained in place and decayed for a period of 45 years from deposition in 1963 to sampling in 2008. Maximum measured activity for ^137^Cs was 27.3 ± 0.6 mBq cm^−3^. Profiles of ^137^Cs activity generally increase with depth from activities of about 2–6 mBq cm^−3^ at the surface to maximum activities at around 8–14 cm depth, followed by a general decrease to values of 0.5–2 mBq cm^−3^ at 30 cm depth.

**Figure 4:**
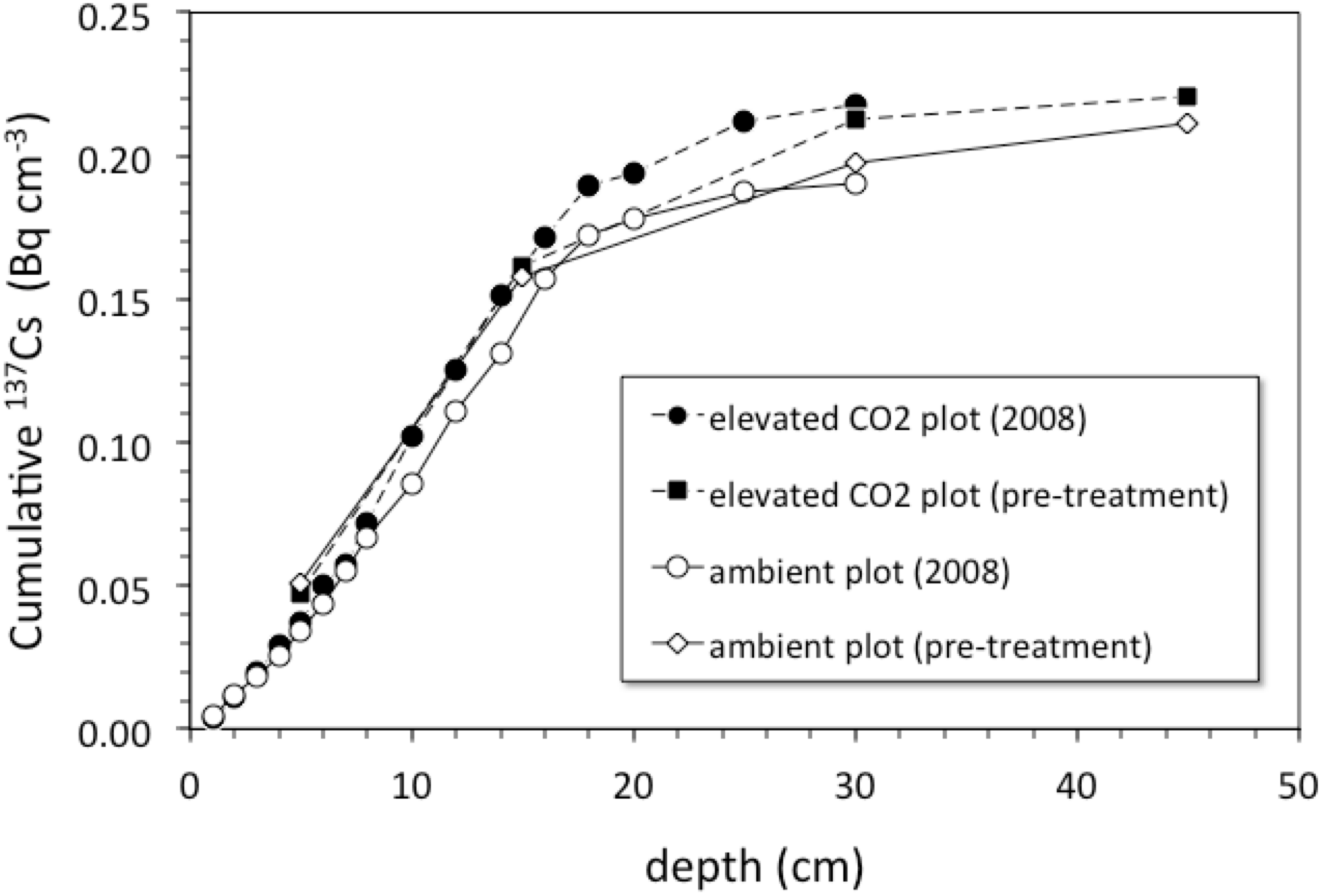
Cumulative ^137^Cs activity. Average cumulative ^137^Cs activity (Bq/cm^2^) vs. depth (cm) in soil cores from ambient and elevated-CO_2_ plots collected in 2008 (after 10 years of CO_2_ release) and for two single sets of samples collected from the same locations in 1997 (before the beginning of CO_2_ release) at the Oak Ridge FACE site.

Pre-treatment core samples for depth ranges of 0–5, 0–15, 15–30, and 30–45 cm collected in 1997 from both the pre-treatment ambient and elevated-CO_2_ plots (prior to initiation of the FACE experiment) had cumulative ^137^Cs activities equal to those of the post-treatment samples collected in 2008 (Fig. 4). There was no measurable activity of ^137^Cs beyond 30 cm depth before and during the FACE experiment (Figs. 3 and 4).

For the 2008 samples, more ^137^Cs activity was found at greater depth in elevated CO_2_ plots when compared to ambient control plots (Fig. 3; *p* < 0.05). There was measureable ^137^Cs activity at the 25–30 cm in soil samples collected in the elevated CO_2_ plots, whereas the average depth of the deepest measurable ^137^Cs activity in the ambient plots was 20.6 ± 2.5 cm. Similar values of ^137^Cs activity were found for the 15–30 cm soil samples collected in 1997.

### Bioturbation model

Bioturbation derived mixing rates were not significantly different between the ambient and elevated CO_2_ plots (Table 2; Fig. 3). We solved Eq. (3) for the biogenic diffusivity (*D*_*b*_), particle advection velocity (*ν*), and feeding rate constant (*γ*) values. Advection velocities are indistinguishable for both treatments with an average value of 0.18–0.19 cm yr^−1^ (Table 2). The feeding rate constants were 0.008 ± 0.003 yr^−1^ for ambient and 0.005 ± 0.001 yr^−1^ for elevated-CO_2_ plots. The *D*_*b*_ values were at 0.53 ± 0.20 cm^2^ yr^−1^ at ambient and 0.63 ± 0.29 cm^2^ yr^−1^ at elevated-CO_2_. These biogenic diffusion coefficients (*D*_*b*_) were used for calculating mixing time constants (*τ*) for a soil layer thickness *L* = 20 cm ($\tau ={L}^{2}{D}_{b}^{-1}$) (Kaste, Heimsath & Bostick, 2007). The top 20 cm layer of soil at the ORNL FACE site has estimated average mixing times ranging from about 640 to 750 years (Table 2) but with wide spatial variability.

### ^210^Pb profiles

**Figure 5:**
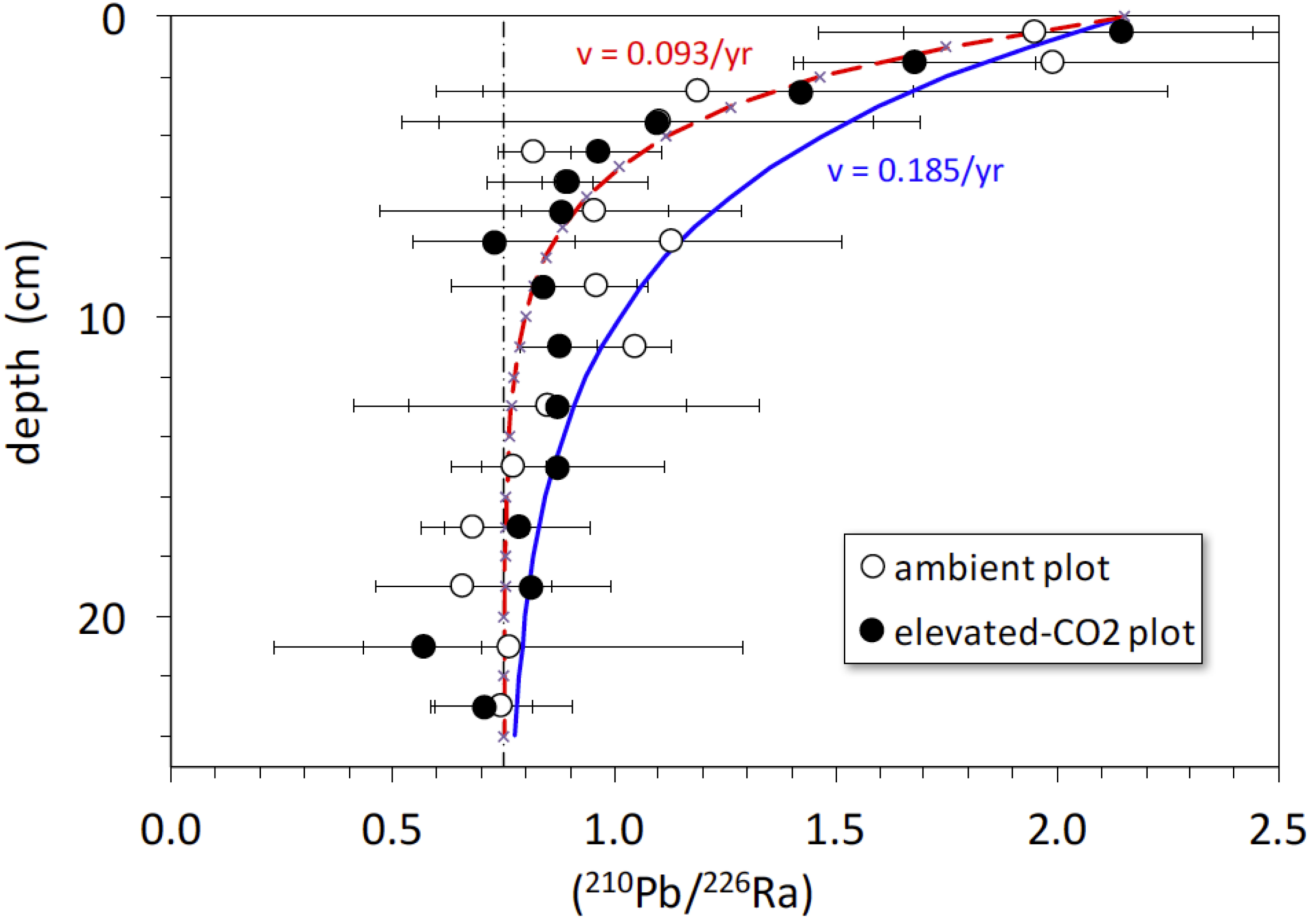
Activity ratio (^210^Pb/^226^Ra) vs. depth (cm) in soil cores collected in 2008 from ambient (open circles) and elevated-CO_2_ (filled circles) plots at the Oak Ridge FACE site. Solid curve (blue) represents constant addition of ^210^Pb to the surface, an advection rate of 0.185 cm yr^−1^ based on best-fit of advection-diffusion model (Eq. (3)) to mean ^137^CS profiles, and decay of ^210^Pb to a steady-state value of 0.75 × (^226^Ra), representing 25% loss of *in situ* ^222^Rn production. Dot-dashed vertical line represents the typical mean value of 0.75 for soil (^210^Pb/^226^Ra) (Graustein & Turekian, 1990). Dashed curve (red) represents constant addition of ^210^Pb to the surface and an advection rate of 0.0.093 cm yr^−1^ based on the best-fit of advection-diffusion model (Eq. (4)) to mean unsupported-^210^Pb profiles, and decay of ^210^Pb to a steady-state value of 0.75 × (^226^Ra). Apparent deficiency of excess ^210^Pb in the soil profiles is consistent with diffusive escape of ^222^Rn produced *in situ*, possibly enhanced by bioturbation and transpiration occurring in the shallow root zone.

The activity ratio (^210^Pb/^226^Ra) is a good indicator of excess (or deficient) ^210^Pb relative to that expected from secular equilibrium with ^226^Ra (at secular equilibrium, (^210^Pb/^226^Ra) = 1). The average (^210^Pb/^226^Ra) activity ratio profiles in the ambient and elevated-CO_2_ plots are similar, showing excess ^210^Pb in the upper 5-to-10 cm and a deficit of ^210^Pb below 10 cm depth (Fig. 5). Best-fit solutions of Eq. (4) to the (^210^Pb/^226^Ra) profiles all yielded lower values of diffusion constant (near 0) and advection rate (∼0.9 cm yr^−1^) than did the ^137^Cs models. We show the best-fit steady-state advection-decay model in comparison with the mean (^210^Pb/^226^Ra) profiles in Fig. 5. The parameters in this model were a constant initial (^210^Pb/^226^Ra) value of 2.3 and a steady-state (^210^Pb/^226^Ra) ratio of 0.75 at depths below 20-to-24 cm, where excess ^210^Pb has decayed to <2% of its initial amount (Fig. 5). The steady-state value of 0.75, representing a 25% loss of *in situ* ^222^Rn production, is based on a survey of ^222^Rn loss in 119 soil cores from undisturbed landscapes in North America. As with the ^137^Cs profiles, no significant difference in mean (^210^Pb/^226^Ra) profiles is evident between the ambient and elevated CO_2_ plots.

## Discussion

**Figure 6:**
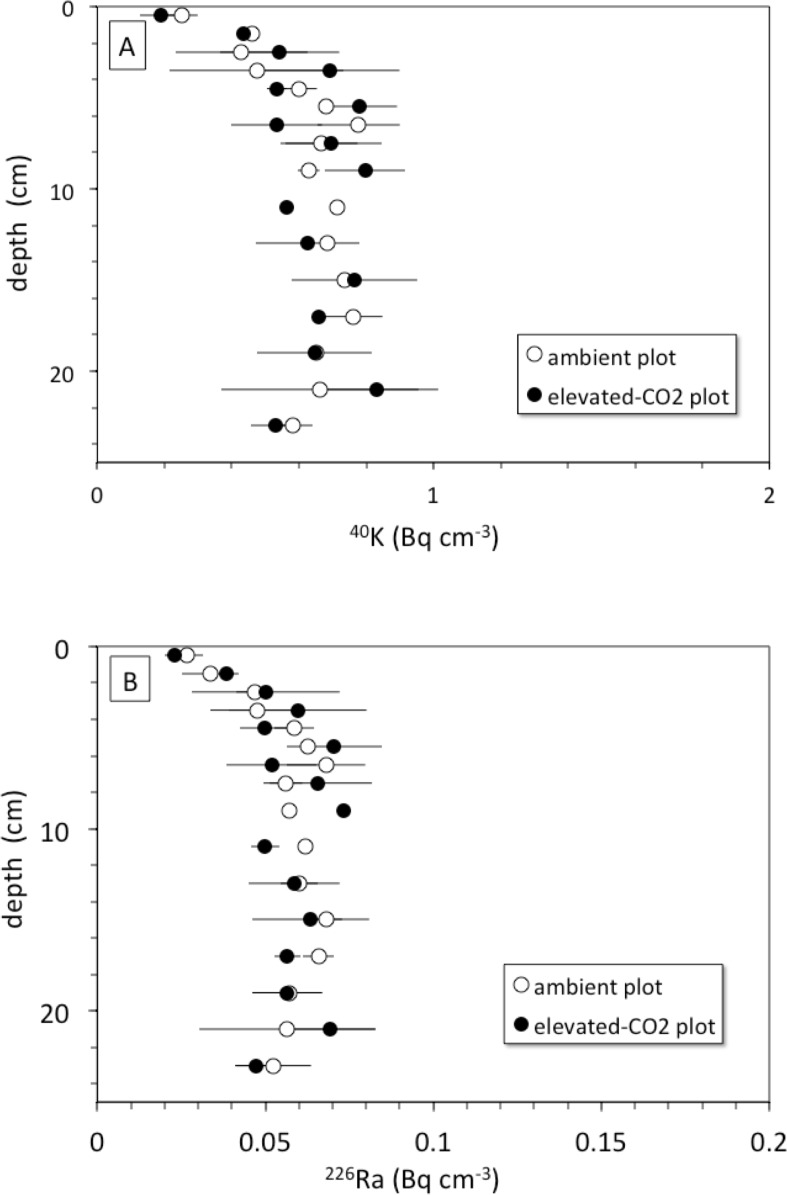
(A) Depth (cm) vs. ^40^K activity (Bq cm^−1^) and (B) depth (cm) vs. ^226^Ra activity (Bq cm^−1^) in average soil profiles from the ambient (open circles) and elevated-CO_2_ (filled circles) plots at the Oak Ridge FACE site.

The rate of bioturbation, as indicated by the best-fit biodiffusion coefficient *D*_*b*_ from the ^137^Cs model (Eq. (3)), was indistinguishable between ambient and elevated CO_2_ conditions (Table 2) despite increased root growth and enhanced earthworm density at the treatment sites (Iversen, Ledford & Norby, 2008; Sánchez-de León et al., 2014). This mixing rate was sufficient to move some SOC from the surface to depth and vice-versa during the 10-year FACE experimental period, suggesting that not all the ^13^C depleted C seen at a given depth at elevated CO_2_ is solely derived from C inputs at that depth. This downward movement of FACE-labeled C by bioturbation may partly contribute to the inability to detect relative increases in SOC below 5 cm at elevated CO_2_ conditions when compared to ambient (Jastrow et al., 2005). Radionuclide profiles of ^40^K and ^226^Ra, however, do not show evidence of more rapid leaching of cations at elevated CO_2_ conditions when compared to the ambient ones, at least in the top 30 cm of soil (Fig. 6). The ^40^K and ^226^Ra profiles rather may indicate decomposition of labile SOC in the upper 5 cm of soil, with corresponding enrichment of ^40^K and ^226^Ra in the residual, more refractory organic matter (Kaste et al., 2011).

The ^137^Cs and unsupported ^210^Pb profiles of this forest resemble those observed in other studies of these radionuclides in soils (Dörr & Münnich, 1989; Dörr & Münnich, 1991; Kaste et al., 2011; Matisoff & Whiting, 2012). Elevated atmospheric CO_2_ results in soils having substantially higher root biomass (Matamala et al., 2003; Lynch et al., 2013), soil CO_2_ concentrations and flux (Taneva et al., 2006; Duval et al., 2013), and in some instances increased microbial and soil macrofaunal activity, including that of earthworms (Sánchez-de León et al., 2014; Sánchez-de León et al., 2018). All these factors could enhance the vertical movement of ^137^Cs and unsupported ^210^Pb within the soil profile under elevated CO_2_ conditions. However, the higher microbial activity in the organic rich soil layers (<15 cm for these soils) often seen in response to elevated CO_2_ conditions ( Gonzalez-Meler & Taneva, 2011; Cheng et al., 2014), could also increase the retention of ^137^Cs and unsupported ^210^Pb in the top layer of the soil (Bruckmann & Wolters, 1994). Indirect evidence supports the notion of potentially higher bioturbation in a higher CO_2_-world. For instance, the feeding constant rate (Table 2) is additive with the ^137^Cs decay constant in Eq. (3) and thus may indicate some net removal of ^137^Cs from the profile by leaching or by faunal or root uptake. These biological transport processes also have consequences for carbon burial at centurial time scales that need to be considered in models of the C cycle.

**Table 2:** Bioturbation. Parameter values obtained from advection-diffusion model. Parameters *D*_*b*_, *v*, and *γ* are derived from best-fits of average ^137^Cs activity profiles to Eq. (2). The soil mixing time *τ* is calculated for *L* = 20 cm. Values are averages of two ambient CO_2_ and two elevated CO_2_ rings ± standard deviations.

**Symbol**	**Parameter**	**Unit**	**Ambient CO_2_**	**Elevated CO_2_**
***D***_***b***_	Bio-diffusion coefficient	cm^2^ yr^−1^	0.53 ± 0.20	0.63 ± 0.29
***v***	Advection term	cm yr^−1^	0.19 ± 0.02	0.18 ± 0.03
*γ*	Feeding rate constant	yr^−1^	0.008 ± 0.003	0.005 ± 0.001
*τ*	Soil mixing time (*τ* = *L*^2^*Db*^−1^)	yr	750 ± 210	640 ± 200

The net long-term rate at which soil material is moved downward by burial and advective transport is given by the model parameter *ν* (from ^137^Cs models this is 0.18 cm yr^−1^, Table 2, but a value of only about $ \frac{1}{2} $ that is indicated by the average unsupported ^210^Pb profiles). Other studies have shown that ^137^Cs transport is faster and somewhat decoupled from that of ^210^Pb (Dörr & Münnich, 1989; Dörr & Münnich, 1991). Over the 10-year duration of the FACE experiment from its initiation in 1998 through our sample collection in 2008, material deposited at the surface (where bulk density is the smallest, Table 1) could be transported by advection to a mean net depth of 1.8 cm. Litter deposited at the surface in the elevated-CO_2_ profiles should be clearly distinct in terms of its *δ*^13^C value, because the CO_2_ released during the FACE experiment had a much lower *δ*^13^C value than that of atmospheric CO_2_ (Fig. 2). In fact, the top 2 cm of the elevated-CO_2_ profile clearly has significantly lower *δ*^13^C values than the ambient profile (Fig. 1B), and much higher SOC content as well (Fig. 1A). These differences are attributable to the influence of the elevated CO_2_ treatment during the FACE experiment. The data shown in Fig. 2 indicate, however, that the influence of the ^13^C-depleted CO_2_ released to the atmosphere at the FACE site appears to have affected the amount and isotopic composition of bulk SOC throughout essentially the entire 30-cm depth of the elevated-CO_2_ soil profile. This implies that other processes must have acted to increase inputs and transport of some fraction of SOC downward at a rate higher than that given by the mean net advective transport term in the bioturbation model. Such processes may include bioturbation caused by higher root growth and turnover as well as the feeding activity of burrowing organisms, and advective transport of dissolved inorganic carbon (DIC), dissolved organic carbon (DOC), and particulate organic carbon (POC) in soil pore water, all of which can accelerate the movement of SOC along specific pathways.

Is there an enhancement of weathering activity at elevated CO_2_? The difference in cumulative ^137^Cs activity between the ambient and elevated CO_2_ plots was apparently present before the CO_2_ experiment started, because it is also seen in the pretreatment soil samples (Fig. 3). Unfortunately, the pretreatment soil samples only indicate total ^137^Cs to a 30 cm depth but not its distribution along the entire soil profile with the deepest sample being 15–30 cm deep. Based on the ^137^Cs profile alone, it cannot be ruled out that the CO_2_ treatment had an effect on the maximum depth of ^137^Cs activity. The importance of earthworms in bioturbation, and for increasing soil porosity and permeability, has been noted in a number of other studies involving the interpretation of ^137^Cs profiles of soils (Bunzl, 2002b; Jarvis et al., 2010; Müller-Lemans & Van Dorp, 1996; VandenBygaart et al., 1998). The increases in root, soil flux and earthworm activity seen in the elevated CO_2_ treatment at this site are consistent with this potential effect of CO_2_ on maximum depth of ^137^Cs. Transport of ^137^Cs could be enhanced by increases in porosity and permeability of soil caused by earthworm activity, which could increase the rate and volume of fluid flow through the soil.

Another mechanism for increasing downward transport of both ^137^Cs and C could be the acidification of soils because of enhanced soil metabolic activity often seen at elevated CO_2_ (e.g.,Taneva et al., 2006; Hopkins et al., 2013). Increases in soil partial pressure of CO_2_ (pCO_2_) in plots exposed to elevated CO_2_ may decrease soil pH and alter metal chemistry (Natali et al., 2008). Lower pH caused by higher *p*CO_2_ can increase soil weathering rate along with desorption of adsorbed cations, production of bicarbonate by carbonic acid neutralization, and consequently enhanced advective transport of desorbed cations and DIC deeper into the soil profile (Andrews & Schlesinger, 2001). The depth profiles of endogenous soil ^40^K and ^226^Ra species show nearly constant concentration of each nuclide with depth in both the ambient and elevated-CO_2_ plots (Fig. 6). This may indicate that the change in soil pH associated with elevated CO_2_ was not sufficient to cause a substantial increase in weathering rate and mobilization of K^+^ and Ra^2+^ ions within the top 30 cm of the soil profile. These two ions could be mostly incorporated within mineral grains, whereas ^137^Cs is associated with mineral surfaces and is therefore more susceptible to desorption (Dörr & Münnich, 1989; Dörr & Münnich, 1991). However, the results presented here do not provide evidence of increased weathering rates in the top 30 cm in response to elevated CO_2_ as suggested elsewhere (Cheng et al., 2010).

### Soil mixing dynamics

There is a rapid decrease in (^210^Pb/^226^Ra) ratios in the ambient and elevated-CO_2_ plots at the FACE site, relative to that predicted by the simple ^210^Pb advection-decay model (Fig. 5). The constant deposition of ^210^Pb from the atmosphere to the soil surface creates a condition of radioactive disequilibrium where ^210^Pb in the shallow parts of soil profiles is in excess of that produced *in situ* by decay of ^226^Ra and intermediate daughters. The profile of excess ^210^Pb can be modeled in terms of soil or sediment accumulation and erosion rates and mixing parameters (Olsen et al., 1981; Robbins, 1986; Kaste, Heimsath & Bostick, 2007; Kaste et al., 2011; Matisoff & Whiting, 2012). The ^210^Pb/^226^Ra profiles depicted in Fig. 5 decay too rapidly with depth to be consistent with simple downward advection at 0.18 cm yr^−1^ and radioactive decay of ^210^Pb. Using Eq. (4), we obtained a best-fit value for advective transport (*v*) of about 0.9 cm yr^−1^. The relatively low and constant value of the ^210^Pb/^226^Ra activity ratio at depth indicates diffusive escape of ^222^Rn (likely via transpiration stream and soil porosity) that was produced *in situ* from ^226^Ra decay. This ^222^Rn escape is possibly enhanced by bioturbation and transpiration occurring in the shallow root zone (top 10 cm) where the bulk density is the lowest (Table 1). The ^137^Cs profiles and the rapid decrease in excess ^210^Pb suggest a distinct soil boundary at about 4 cm deep, below which most of the ^137^Cs activity resides (Fig. 5). This 0–4 cm depth soil layer is also evident in isotope profiles shown in Figs. 1 and 6. This soil multi-isotope boundary at 4 cm depth is consistent with the enhanced SOC accumulation at elevated CO_2_ conditions when compared to ambient seen at the site between 0 and 5 cm (Fig. 1 of Jastrow et al., 2005). Further, these results are also consistent with the presence at the site of endogeic earthworms (Sánchez-de León et al., 2018), which avoid the soil surface likely preventing predation or competition with litter layer fauna.

A second soil isotope boundary is detected at about 16 cm deep, where the ^210^Pb/^226^Ra activity ratio approaches the typical disequilibrium value of 0.75 seen in deeper soils (Graustein & Turekian, 1990) (Fig. 5). This 4–16 cm soil section may represent a soil mixing compartment influenced by root proliferation and earthworm activity, potentially redistributing and homogenizing SOC concentration within this depth range (Fig. 1A). This may partly prevent detection of soil C accrual in response to elevated CO_2_ levels at these depths (Fig. 1A), despite the isotopic evidence for input of new SOC (Fig. 1B). More research using multiple radioisotope tracers to detect soil profile mixing sections may allow better determinations of C dynamics than are possible by using the traditional arbitrary depth comparisons.

## Conclusion

The ^137^Cs profile and the associated bioturbation models represent a 45-year period between the deposition of the ^137^Cs bomb-spike in 1963 and the collection of the soil cores in 2008. The parameter values obtained from the advection-diffusion models are not significantly different between the average ambient and elevated-CO_2_ profiles (Table 2). Elapsed time from the beginning of the FACE experiment to the time of sampling was inadequate to cause a substantial response in terms of observable soil bioturbation. Future studies require greater sample density to acquire sufficient statistical evidence for observing subtle changes in such heterogeneous systems. However, even during the decade-long duration of the FACE experiment there is significant evidence for increased inputs of new SOC at soil depth and for enhanced migration of SOC beyond 20 cm depth, likely caused by both biological and chemical processes related to elevated CO_2_. The multiple isotopic tracer approach used here indicated at least two soil compartments: one in the top 4 cm where C accumulated in response to CO_2_ and one below 4 cm where SOC may have been redistributed. These “soil mixing cells” may bring into question the traditional depth comparisons for SOC (e.g., 0–5 cm, 0–10 cm) that are routinely done in soil studies, and which may obscure detection of SOC changes in response to environmental factors.

The biological and geochemical effects of elevated atmospheric CO_2_ could have substantial consequences for carbon burial and the fate of deep soil C over a longer time scale (centuries) than usually considered in soil C and Earth System models (Schmidt et al., 2011; Kaste et al., 2011; Todd-Brown et al., 2013). For instance, in a typical 100–200-year model run, C residing in a given soil layer could move downward by bioturbation and weathering to another soil layer depicted in a model (e.g., CLM4.5), affecting rates of decomposition, soil C turnover times and the soil feedback on the atmospheric concentration of CO_2_. Bioturbation processes could substantially increase the net long-term storage of soil C and should be incorporated in soil-atmosphere interaction models.

##  Supplemental Information

10.7717/peerj.5356/supp-1 Supplemental Information 1 Raw data for radioisotopes and soil bulk density Raw data for ^137^Cs, ^210^Pb, ^40^K, ^226^Rn, and soil bulk density under elevated CO_2_ concentration.

10.7717/peerj.5356/supp-2 Supplemental Information 2 Raw data for carbon percentage (%) and carbon isotope ratio (d13C/d12C) Raw data for concentration of carbon (%) in soil and carbon isotope ratio (d13C/d12C) in soil under elevated CO_2_ concentration.

## Additional Information and Declarations

### Data Availability

The following information was supplied regarding data availability: The raw data are provided in the Supplemental Files.
